# Anticipated reactions to learning Alzheimer’s disease biomarker results

**DOI:** 10.1186/s13195-022-01027-2

**Published:** 2022-06-22

**Authors:** Lindsay R. Clark, Claire M. Erickson, Erin M. Jonaitis, Yue Ma, Nathaniel A. Chin, Kristin Basche, Frederick B. Ketchum, Carey E. Gleason

**Affiliations:** 1 Division of Geriatrics and Gerontology, Department of Medicine, University of Wisconsin-Madison School of Medicine and Public Health, 600 Highland Avenue, Madison, WI USA; 2 Geriatric Research Education and Clinical Center, William S. Middleton Memorial Veterans Hospital, 2500 Overlook Terrace, Madison, WI USA; 3 Neuroscience & Public Policy Program, University of Wisconsin-Madison School of Medicine and Public Health, 600 Highland Avenue, Madison, WI USA; 4 Department of Neurology, University of Wisconsin-Madison School of Medicine and Public Health, 600 Highland Avenue, Madison, WI USA

**Keywords:** Preclinical Alzheimer’s disease, Risk communication, Biomarker disclosure, Psychometrics, Psychosocial

## Abstract

**Introduction:**

We developed the Alzheimer’s Biomarker Survey to assess willingness to enroll in biomarker studies that disclose results and anticipated reactions to an elevated biomarker result.

**Methods:**

Participants included cognitively unimpaired adults enrolled in longitudinal AD studies (*n* = 334, mean age = 64.8 ± 7.7, 44% non-Hispanic Black or African American). Exploratory and confirmatory factor analyses determined the latent structure comprising anticipated reactions to learning AD biomarker results. Measurement invariance was tested across racial groups.

**Results:**

Two models comprising behavior change and psychological impact fit well for the total sample and the two racial groups. The 2-factor *behavior change* model assessed constructs of planning and dementia risk-reduction. The 3-factor *psychological impact* model assessed constructs of distress, cognitive symptoms, and stigma. Both models exhibited measurement invariance across racial groups.

**Discussion:**

The 28-item Anticipated Reactions to AD Biomarker Disclosure scale is a reliable and valid measure of anticipated reactions when communicating AD biomarker results to research participants.

## Background

Recent FDA approval of the first disease-modifying therapy for AD [1] and development of blood-based biomarkers [2] increase the likelihood that adults with mild symptoms or at-risk asymptomatic adults will learn their AD biomarker results. Shifting to a biomarker-based diagnosis may change the experience of living with AD [3]. Stigma associated with AD dementia may spill over to prodromal phases, resulting in negative psychosocial outcomes and reduced willingness to seek care [3]. However, learning biomarker results in preclinical or prodromal stages may have positive outcomes such as advanced care planning and reducing modifiable risk factors for dementia [4]. To develop best practices and toolkits for AD biomarker disclosure, information is needed about research participants’ perspectives and likely reactions following disclosure.

A few studies have assessed interest or attitudes towards learning biomarker-based risk results or enrolling in AD clinical trials in which biomarker test results may be disclosed. One study found experience with AD increased interest in results disclosure, but learning about potential limitations of results reduced interest levels [5]. Further, interest was overall lower for the general population sample compared to research participants. Another study focusing on the desire to know AD risk information and effects on life plans found AD research participants reported a desire to know these results to contribute to research, arrange personal affairs, prepare family for illness, and move life plans closer into the future [6]. Focus groups have similarly found that interest may wane when limitations of biomarker results are understood [7], that desire to learn results varies across stakeholder groups (e.g., healthy elderly, caregivers, nursing staff, researchers, clinicians) [8], and that participants discuss both positive and negative potential impacts of learning biomarker results [8]. Results from the above studies provide common themes for interest in participation in AD research studies that disclose results, and the rationale behind these decisions. Many of the studies, though, included either small samples or lacked samples diverse in age, education, race/ethnicity, or socioeconomic status. Lack of inclusion of participants from historically under-represented groups, importantly, contributes to ongoing health disparities in AD biomarker research and clinical trials, ultimately limiting generalizability of treatment access and efficacy. Additionally, although prior studies have provided information on reasons for learning biomarker or other risk-related results, no studies have specifically assessed anticipated reactions to learning positive (i.e., high-risk) biomarker results.

We developed a telephone survey to assess willingness to participate in AD biomarker studies that disclose results, and anticipated reactions following disclosure. We also assessed additional contextual factors that may predict willingness or potential reactions including experience with or concerns about AD, perceived health and memory, health locus of control, brain health social norms, self-ratings of memory and health, research attitudes, and perceived discrimination. We administered this survey to participants enrolled in the Wisconsin Alzheimer’s Disease Research Center (ADRC) or Wisconsin Registry for Alzheimer’s Prevention (WRAP) studies. To ensure inclusion of survey responses from participants from under-represented groups, we focused recruitment efforts to bolster participation of individuals self-identifying as Black or African American, who constituted about half our sample. In this paper, we present information on overall survey development, reliability (internal consistency), and validity (factor structure) of the “Anticipated reactions to learning AD biomarker results” scale, and its measurement invariance across racial groups.

## Methods

### Survey development

The study team consulted with the University of Wisconsin-Madison Survey Center (UWSC) to develop the survey. First, scales measuring constructs of interest were reviewed from the existing literature. Items pulled from existing scales assessing experience with and concern about AD [9], perceived health, health locus of control [10], brain health social norms [11], research attitudes [12], and chronic experiences of perceived discrimination due to race, ethnicity, gender, age, or other interpersonal characteristics [13] were included and modified for telephone administration. New items were developed to assess willingness to learn biomarker results and potential positive or negative reactions to learning hypothetical positive/high-risk biomarker results. Five hypothetical scenarios assessed willingness to participate in biomarker studies with or without results disclosure on a 5-point Likert scale (response options not-at-all-willing to extremely-willing). These items included a general AD biomarker study and items that varied by specific biomarker collection method (PET, lumbar puncture, blood draw). Two open-ended questions assessed reasons for response and any concerns about the biomarker study with results disclosure. Eight items on reasons for learning amyloid PET results were adapted from an existing questionnaire [14, 15]. Thirty-three items assessed anticipated reactions to learning high-risk (e.g., positive) biomarker results on a 5-point Likert scale. All items also included the option to refuse to provide a response or provide an unknown response; unknown or refused responses were coded as missing values. Internal and external content experts reviewed and modified the survey. Lastly, the survey was modified using feedback from five adults that pilot-tested the survey for duration and understanding of questions.

### Recruitment

Four-hundred participants were recruited from the Wisconsin ADRC and WRAP, two ongoing longitudinal AD-related studies. Inclusion criteria for recruitment included age 45–89, self-identified ethnicity/race as either non-Hispanic Black or African American or non-Hispanic White, and no research or clinical diagnosis of mild cognitive impairment or dementia based on National Institutes on Aging-Alzheimer’s Association diagnostic criteria [16, 17]. We consulted with the UW Community Advisors on Research Design & Strategies (CARDS) to develop our recruitment materials and strategies based on feedback from a community focus group with diverse racial and socioeconomic backgrounds. Eligible participants were mailed a recruitment letter and information sheet and then contacted by phone. If interested in participating, the information sheet was reviewed and oral consent was obtained. Participants were compensated $25 for completing the survey.

### Survey administration

The survey was administered by trained UWSC interviewers using Computer-Assisted Telephone Interview (CATI) from 01/06/2020 to 03/16/2020. The UWSC used CASES 5.6 provided by the Computer-Assisted Survey Methods Program at the University of California-Berkeley. All UWSC telephone interviewers received training (e.g., fundamentals of data collection, observation, role play, mock interviews, ongoing refresher training) and an additional 4 h of project-specific training and practice before data collection. Quality control of the CATI interviewers was implemented through ongoing monitoring by UWSC supervisors. The average survey length was 33 min.

### Statistical analysis

#### Survey response patterns

Descriptive statistics (e.g., means, percentages) were used to provide information on sample characteristics and evaluate response patterns on survey items.

#### Evaluating factor structure of total sample

The Kaiser–Meyer–Olkin (KMO) for checking sampling adequacy and Bartlett’s Test of Sphericity were calculated to assess the suitability of the data for factor analysis. Exploratory factor analysis (EFA) with an oblique rotation was conducted using maximum likelihood estimation on the 41 Likert-scale items assessing reasons for learning results and anticipated reactions to learning high-risk biomarker results. One item (alcohol intake) was excluded from analysis due to missing data (no response for 32 participants). Due to the ordinal structure of the Likert response data, the EFA was conducted using a polychoric correlation matrix [18]. Number of factors was determined based on (1) ≥ 3 salient item loadings (e.g., pattern coefficients ≥ 0.30), (2) high internal consistency (e.g., alpha coefficients ≥ 0.70), (3) model parsimony, and (4) theoretical/clinical meanings. Confirmatory factor analysis (CFA) was next applied to refine and confirm the factor structure identified by EFA. Overall model fit was assessed using fit indices including the comparative fit index (CFI [19]), the root mean squared error of approximation (RMSEA [20]), and the standardized root mean squared residual (SRMR). Model fit was considered adequate by meeting the following criteria: CFI ≥ 0.95, RMSEA ≤ 0.08, and SRMR ≤ 0.08[21–23]. Misfit in individual parameters was evaluated using model modification indices (MI), which are the amount of reduction in the *χ*^2^ model if a parameter fixed at zero or constrained to be equal across groups were freely estimated. MIs for each parameter were examined and parameters were freed in cases in which the MIs were exceptionally high (e.g., MI > 20). The EFA was conducted using the R Psych package [24] and the CFA was conducted using the R Lavaan package [25]. Respondents with missing data were excluded from the EFA. Respondents with missing data were included in the CFA using maximum likelihood estimation with robust standard errors (Lavaan MLR estimator) to account for missing data.

#### Testing measurement invariance across racial groups

The final CFA model was first tested for each racial group separately. If the CFA fit well for both groups, measurement invariance was next tested with a hierarchical series of nested two-group CFA models testing in the following order: (1) configural invariance (baseline model and implies that the same latent constructs are measured for both groups), (2) metric variance (item/indicator loadings onto latent constructs are equal across groups), and (3) scalar invariance (item/indicator intercepts are equal across groups). Scalar invariance is required to confirm measurement invariance and allows meaningful comparison in the latent constructs between groups. At each stage, model fit was assessed using the criteria above (CFI ≥ 0.95, RMSEA ≤ 0.08, and SRMR ≤ 0.08), as well as using a change in CFI < 0.01 to determine if each level of invariance was confirmed (e.g., configural vs metric)[26]. Ordinal CFA models were not estimable with the available sample size; therefore, to accommodate nonnormality, models were estimated using the maximum likelihood estimator with robust standard errors (MLR). Missing data were accommodated using full information maximum likelihood.

## Results

### Sample characteristics

The overall response rate for the survey was 83.5% (81.3% for Black respondents; 85.3% for White respondents; *n* = 334) (Table 1). Of the 66 participants who were mailed recruitment letters but did not enroll, 13 declined participation, 5 were unable to participate due to physical or cognitive limitations, and 48 were unable to be reached. Participants were on average 65 years old (range: 45–85), more likely to be women (74%), and highly educated (58% with a Bachelor’s degree). One hundred forty-eight participants (44%) self-identified as non-Hispanic Black or African American and 186 participants (56%) self-identified as non-Hispanic White. A majority had a family history of dementia (62%) and/or had cared for a person with dementia (68%).

**Table 1:** Sample characteristics (*N* = 334)

Characteristic^a^	
Age (mean, SD)	64.8 (7.7)
Gender (*n*, %)	
*Women*	248 (74%)
*Men*	86 (26%)
Education (*n*, %)	
≥ *Bachelor’s degree*	195 (58%)
< *Bachelor’s degree*	139 (42%)
Race (*n*, %)	
*Black or African American*	148 (44%)
*White*	186 (56%)
Recruitment source (*n*, %)	
*Wisconsin ADRC*	167 (50%)
*WRAP*	167 (50%)
Family history of dementia (*n*, %)	
*Positive*	208 (62%)
*Negative*	125 (37%)
Dementia caregiver (ever) (*n*, %)^b^	
*Yes*	228 (68%)
*No*	86 (26%)
Research attitudes (RAQ-7), (mean, SD; range)	29.9 (3.5; 20–35)
Perceived discrimination, (mean, SD; range)	16.5 (6.4; 7–37)

^a^All characteristics were based on self-report; ^b^Data missing for *n* = 20 (6%) participants

### Anticipated reactions to learning AD biomarker results

Table 2 displays response distributions to reasons for learning AD biomarker results and anticipated reactions to learning elevated biomarker results. Reasons rated most often as “very” or “extremely” important for learning AD biomarker results included informing lifestyle changes to help prevent Alzheimer’s (83% respondents) and reducing personal worry if results did not indicate a higher risk for developing Alzheimer’s (80% respondents). If biomarker results were high (i.e., indicating higher risk for AD dementia), about 40% of respondents would feel “very” or “extremely” upset, sad, anxious or nervous, a loss of personal control, and would likely seek mental health services. Twenty-two to 25% expressed concerns about the possibility their doctor, family, friends, or co-workers would act differently toward them. However, 79% indicated they would be likely to tell a loved one their results and 91% felt their family would be supportive. Few participants indicated they would have problems enjoying life because of their results (17%), feel bad about themselves or that they are a failure (5%), or have thoughts they would be better off dead or consider hurting themselves (4%). Approximately 80% responded they would be likely to make changes to diet or exercise, and engage stress reduction activities or mental/cognitive activities if they learned their biomarker results were high.

**Table 2:** Anticipated reactions to AD biomarker disclosure

Item	Not at all [1] *N* (%)	A little [2] *N* (%)	Somewhat [3] *N* (%)	Very [4] *N* (%)	Extremely [5] *N* (%)	Mean (SD)
Prompt: *I’m going to tell you some reasons why someone might want to learn their brain marker results. Please rate how important each of these are to you, as a reason to learn your brain marker results*						
To know more about your risk of developing Alzheimer’s	18 (5.4)	27 (8.2)	55 (16.6)	91 (27.5)	140 (42.3)	3.93 (1.2)
To inform lifestyle changes you might make, such as diet or exercise that might help prevent Alzheimer’s	9 (2.7)	10 (3.0)	38 (11.4)	105 (31.4)	172 (51.5)	4.26 (1.0)
To put your mind at ease if you found out your brain marker results did not indicate a higher risk for developing Alzheimer’s	15 (4.5)	11 (3.3)	41 (12.3)	115 (34.4)	152 (45.5)	4.13 (1.0)
To be able to participate in clinical trials attempting to lower brain marker results	13 (3.9)	16 (4.8)	68 (20.5)	126 (38.0)	109 (32.8)	3.91 (1.0)
To arrange your personal affairs, such as insurance, your will, or your finances	32 (9.6)	21 (6.3)	69 (20.7)	94 (28.1)	118 (35.3)	3.73 (1.3)
To confirm the feeling that you might already be developing symptoms of Alzheimer’s	28 (8.4)	24 (7.2)	61 (18.3)	105 (31.5)	115 (34.5)	3.77 (1.2)
To prepare your family for your possible illness in the future	18 (5.4)	16 (4.8)	54 (16.2)	108 (32.3)	138 (41.3)	3.99 (1.1)
A desire to start doing things sooner than you had planned	24 (7.2)	21 (6.3)	65 (19.5)	121 (36.2)	103 (30.8)	3.77 (1.2)
Prompt: *I want you to imagine that you participated in a study and were told that your brain marker results were high. While these results do not necessarily mean you will develop Alzheimer’s, it means you have a higher risk of developing Alzheimer’s. If you learned that your brain marker results were high and you were at higher risk of developing Alzheimer’s…*						
How upset would you feel about your results?	25 (7.5)	50 (15)	125 (37.5)	82 (24.6)	51 (15.3)	3.25 (1.1)
How sad would you feel about your results?	12 (3.6)	50 (15)	125 (37.5)	87 (26.1)	59 (17.7)	3.39 (1.1)
How anxious or nervous would you feel about your results?	28 (8.4)	49 (14.7)	126 (37.8)	88 (26.4)	42 (12.6)	3.20 (1.1)
How relieved would you feel about your results?	96 (29.1)	43 (13.0)	83 (25.2)	72 (21.8)	36 (10.9)	2.72 (1.4)
How happy would you feel about your results?	202 (61.2)	27 (8.2)	47 (14.2)	30 (9.1)	24 (7.3)	1.93 (1.3)
How worried would you be about your risk of developing Alzheimer’s?	18 (5.4)	49 (14.7)	120 (35.9)	94 (28.1)	53 (15.9)	3.34 (1.1)
How uncertain would you be about what your results mean about your risk of developing Alzheimer’s?	31 (9.3)	49 (14.8)	156 (47.0)	76 (22.9)	20 (6.0)	3.02 (1.0)
How uncertain would you be about what your results mean for your children or family’s risk of Alzheimer’s?	31 (9.3)	34 (10.2)	105 (31.6)	108 (32.5)	54 (16.3)	3.36 (1.2)
How frustrated would you feel about there being no definite Alzheimer’s prevention guidelines for you?	40 (12.0)	56 (16.8)	90 (27.0)	89 (26.7)	58 (17.4)	3.21 (1.3)
How hopeless would you feel about the future?	89 (26.6)	73 (21.9)	112 (33.5)	39 (11.7)	21 (6.3)	2.49 (1.2)
How concerned would you feel about how your results might affect your insurance status?	81 (24.3)	29 (8.7)	92 (27.7)	79 (23.8)	51 (15.4)	2.97 (1.4)
How concerned would you feel about the possibility that your doctor might learn the results and treat you differently?	145 (43.5)	49 (14.7)	65 (19.5)	46 (13.8)	28 (8.4)	2.29 (1.4)
How concerned would you be that if others, such as family, friends, or co-workers, learned the results, they would act differently toward you?	87 (26.0)	57 (17.1)	107 (32.0)	55 (16.5)	28 (8.4)	2.64 (1.3)
How likely would you be to tell a loved one your results?	11 (3.3)	16 (4.8)	44 (13.2)	130 (38.9)	133 (39.8)	4.07 (1.0)
How difficult would it be to talk about your results with family members?	142 (42.6)	57 (17.1)	80 (24.0)	44 (13.2)	10 (3.0)	2.17 (1.2)
How supportive do you feel that your family would be?	2 (0.6)	4 (1.2)	23 (6.9)	138 (41.6)	165 (49.7)	4.39 (0.7)
How likely is it that you would feel a loss of personal control?	38 (11.4)	49 (14.8)	117 (35.2)	80 (24.1)	48 (14.5)	3.15 (1.2)
How likely is it that you would have problems enjoying life because of your results?	104 (31.1)	77 (23.1)	96 (28.8)	38 (11.4)	18 (5.4)	2.37 (1.2)
How likely is it that you would regret getting your results?	169 (50.8)	54 (16.2)	66 (19.8)	34 (10.2)	10 (3.0)	1.98 (1.2)
How likely is it that you would feel bad about yourself, or that you are a failure or have let yourself or your family down?	242 (72.9)	52 (15.7)	21 (6.3)	9 (2.7)	8 (2.4)	1.46 (0.9)
How likely is it that you would have thoughts that you would be better off dead or consider hurting yourself in some way?	272 (82.2)	30 (9.1)	17 (5.1)	5 (1.5)	7 (2.1)	1.32 (0.8)
How likely is it that you would seek mental health services?	56 (16.9)	53 (16.0)	84 (25.3)	84 (25.3)	55 (16.6)	3.09 (1.3)
How likely is it that you would have more trouble concentrating on things, such as reading the newspaper or watching television?	124 (37.5)	66 (19.9)	96 (29.0)	30 (9.1)	15 (4.5)	2.23 (1.2)
How likely is it that you would have more trouble remembering things, such as recent conversations or events?	116 (35.2)	61 (18.5)	91 (27.2)	41 (12.4)	21 (6.4)	2.36 (1.3)
How likely is it that you would be less confident about your ability to carry out daily activities?	99 (29.6)	71 (21.3)	120 (35.9)	30 (9.0)	14 (4.2)	2.37 (1.1)
How likely is it that you would view the length of time you have to live differently?	56 (16.8)	33 (9.9)	89 (26.7)	97 (29.1)	58 (17.4)	3.20 (1.3)
How likely would you be to make changes to your diet?	12 (3.6)	13 (3.9)	43 (12.9)	12 (38.0)	139 (41.6)	4.10 (1.0)
How likely would you be to exercise or do more physical activity?	15 (4.5)	19 (5.8)	36 (10.9)	130 (39.4)	130 (39.4)	4.03 (1.1)
How likely would you be to make changes to your medications?	38 (11.6)	18 (5.5)	67 (20.4)	105 (31.9)	101 (30.7)	3.65 (1.3)
How likely would you be to make changes to or start taking vitamins or herbal supplements?	41 (12.3)	17 (5.1)	60 (18.0)	117 (35.1)	98 (29.4)	3.64 (1.3)
How likely would you be to engage in activities to reduce stress?	14 (4.2)	17 (5.1)	40 (12.0)	147 (44.0)	116 (34.7)	4.00 (1.0)
How likely would you be to engage in mental or cognitive activities such as crossword puzzles or Sudoku?	13 (3.9)	14 (4.2)	35 (10.5)	147 (44.0)	125 (37.4)	4.07 (1.0)
How likely would you be to reduce or eliminate alcohol intake?	34 (11.3)	13 (4.3)	49 (16.2)	102 (33.8)	104 (34.4)	3.76 (1.3)

*Footnote:* Although the response is an ordinal scale, the level indicated by each response category can be considered approximately as an interval scale

### EFA and CFA for the total sample

The Kaiser, Meyer, Olkin (KMO = 0.88) measure of sampling adequacy and Bartlett’s test of sphericity (*χ*^2^(820) = 6117.87, *p* < 0.001) suggested data were appropriate for factor analysis. Twenty-eight participants had missing data for at least one item; this resulted in a total of *n* = 306 complete observations included in the EFA. Initial EFA suggested model structures between 2 and 7 factors. 12 items were removed due to cross-loading onto multiple factors or not loading onto any factors. Subsequent EFA on 28 items resulted in a five-factor structure which met criteria including all indicators loading uniquely onto factors (e.g., no cross-loading, ranged from 0.43 to 0.95), each factor was internally reliable (e.g., Cronbach’s alpha ranged from 0.71 to 0.91), and the resulting factor structure was theoretically/clinically meaningful (Table 3). However, the subsequent CFA conducted on the full sample (*n* = 334) indicated inadequate model fit (CFI = 0.91; RMSEA = 0.06; SRMR = 0.07). Additionally, the correlation pattern (Table 4) between two of the factors and the other three factors suggested these factors comprise two distinct models: (1) Behavior Changes model (2 factors: planning [8 items], dementia risk-reduction [6 items]) and (2) Psychological Impact model (3 factors: distress [8 items], stigma [3 items], cognitive symptoms [3 items]). Therefore, subsequently, separate CFAs were conducted for the Behavior Changes and the Psychological Impact models. CFA results for both scales are presented in Table 5. For the Behavior Changes model, following high modification indices, 2 items (“to be able to participate in clinical trials attempting to lower brain marker results” and “to inform lifestyle changes you might make that might help prevent Alzheimer’s”) were allowed to load on both planning and dementia risk-reduction factors. Further, correlated errors were permitted between items regarding likelihood to make changes to diet and exercise, as well as between items regarding likelihood to make changes to medications and vitamins/herbal supplements. Using this modified model, the final two-factor model for the Behavior Change CFA demonstrated good model fit indices and loadings (CFI = 0.97, RMSEA = 0.05, SRMR = 0.04). For the Psychological Impact model, following high modification indices, correlated errors were permitted between two items about feelings of uncertainty (e.g., uncertain about the meaning of results for self and family), between two items about feeling sad or upset, between two items about feeling upset and anxious/nervous, and between two items about feeling anxious/nervous and frustrated. Using this modified model, the final three-factor model for the Psychological Impact CFA demonstrated good model fit indices and loadings (CFI = 0.97, RMSEA = 0.05, SRMR = 0.05). The final 28-item scale is included in Table 6.

**Table 3:** Exploratory Factor Analysis (EFA) 5 Factor Model Results

	Loading	Alpha
**Preparatory/planning behavior**		.91
To know more about your risk of developing Alzheimer’s	0.78	
To inform lifestyle changes you might make, such as diet or exercise, that might help prevent Alzheimer’s	0.63	
To put your mind at ease if you found out your brain marker results did not indicate a higher risk for developing Alzheimer’s	0.67	
To be able to participate in clinical trials attempting to lower brain marker results	0.62	
To arrange your personal affairs, such as insurance, your will, or your finances	0.80	
To confirm the feeling that you might already be developing symptoms of Alzheimer’s	0.92	
To prepare your family for your possible illness in the future	0.92	
A desire to start doing things sooner than you had planned	0.82	
**Psychological distress**		.90
How upset would you feel about your results?	0.90	
How sad would you feel about your results?	0.88	
How anxious or nervous would you feel about your results?	0.82	
How worried would you be about your risk of developing Alzheimer’s?	0.82	
How uncertain would you be about what your results mean about your risk of developing Alzheimer’s?	0.61	
How uncertain would you be about what your results mean for your children or family’s risk of Alzheimer’s?	0.43	
How frustrated would you feel about there being no definite Alzheimer’s prevention guidelines for you?	0.65	
How hopeless would you feel about the future?	0.61	
**Dementia risk-reducing behavior**		.87
How likely would you be to make changes to your diet?	0.92	
How likely would you be to exercise or do more physical activity?	0.90	
How likely would you be to make changes to your medications?	0.66	
How likely would you be to make changes to or start taking vitamins or herbal supplements?	0.70	
How likely would you be to engage in activities to reduce stress?	0.79	
How likely would you be to engage in mental or cognitive activities such as crossword puzzles or Sudoku?	0.56	
**Subjective cognitive decline**		.86
Have more trouble concentrating on things, such as reading the newspaper or watching television?	0.73	
Have more trouble remembering things, such as recent conversations or events?	0.95	
Be less confident about your ability to carry out daily activities?	0.85	
**Stigma**		.71
How concerned would you feel about how your results might affect your insurance status?	0.54	
How concerned would you feel about the possibility that your doctor might learn the results and treat you differently?	0.80	
How concerned would you be that if others, such as family, friends, or co-workers, learned the results, they would act differently toward you?	0.55	
**Model fit indices**		
CFI	.91	
RMSEA	.06	
SRMR	.07	

Cutoffs for model fit indices: CFI ≥ 0.95, RMSEA ≤ 0.06, SRMR ≤ 0.08

**Table 4:** Exploratory Factor Analysis – correlations among factors

	Factor: planning	Factor: risk-reduction	Factor: distress	Factor: cognitive symptoms	Factor: stigma
Factor: planning		**0.40**	0.09	0.20	0.11
Factor: risk-reduction	**0.40**		0.19	0.13	0.10
Factor: distress	0.09	0.19		**0.37**	**0.35**
Factor: cognitive symptoms	0.20	0.13	**0.37**		**0.29**
Factor: stigma	0.11	0.10	**0.35**	**0.29**	

Inter-factor correlations demonstrate correlations between planning and risk-reduction factors (*r* = 0.40), and correlations among distress, cognitive symptoms, and sigma factors (*r* = 0.29–0.37)

**Table 5:** Standardized factor loadings and model fit indices for confirmatory factor analysis final models

	Total sample (*n* = 334)	Black or African American (*n* = 148)	White (*n* = 184)
Behavior change model			
Dementia risk-reduction factor			
Inform lifestyle changes that might help prevent Alzheimer’s^a^	0.34	0.21	0.43
Participate in clinical trials to lower brain marker results^a^	0.22	0.11	0.30
Changes to diet	0.72	0.62	0.80
Do more physical activity/exercise	0.77	0.78	0.77
Changes to medications	0.63	0.60	0.62
Changes to vitamins or herbal supplements	0.68	0.55	0.71
Engage in activities to reduce stress	0.85	0.85	0.83
Engage in mental or cognitive activities	0.59	0.60	0.59
Planning factor^b^			
Know more about risk of developing Alzheimer’s	0.71	0.75	0.68
Inform lifestyle changes that might help prevent Alzheimer’s^a^	0.51	0.64	0.47
Put mind at ease if you found out your brain marker results did not indicate a higher risk for developing Alzheimer’s	0.67	0.65	0.69
Participate in clinical trials to lower brain marker results^a^	0.54	0.67	0.46
Arrange personal affairs, such as insurance, will, or finances	0.75	0.73	0.75
Confirm feeling already developing symptoms of Alzheimer’s	0.85	0.81	0.86
To prepare family for possible illness in the future	0.89	0.88	0.88
Desire to start doing things sooner than had planned	0.84	0.86	0.81
Model fit^3^			
CFI	.97	.98	.96
RMSEA	.05	.05	.06
SRMR	.04	.04	.06
Psychological impact model			
Distress			
Upset	0.72	0.68	0.75
Sad	0.83	0.82	0.84
Anxious/nervous	0.80	0.78	0.82
Worried about your risk of developing Alzheimer’s	0.81	0.83	0.80
Uncertainty (personal risk of developing Alzheimer’s)	0.63	0.70	0.56
Uncertainty (children/family risk of developing Alzheimer’s)	0.54	0.60	0.54
Frustrated about lack of Alzheimer’s prevention guidelines	0.74	0.76	0.70
Hopeless about the future	0.74	0.74	0.74
Subjective cognitive decline			
Trouble concentrating	0.77	0.72	0.82
Trouble remembering	0.88	0.87	0.88
Less confident about ability to carry out daily activities	0.84	0.86	0.82
Stigma			
Concern about how results might affect insurance status	0.55	0.56	0.53
Concern doctor might learn results and treat you differently	0.65	0.64	0.67
Concern treated differently by others if learn results (family, friends, co-workers)	0.80	0.85	0.73
Model fit^c^			
CFI	.97	.94	.98
RMSEA	.05	.08	.04
SRMR	.05	.06	.05

^a^Items cross-loaded on both factors

^b^Items adapted from “Views and Perceptions of Amyloid Imaging” Questionnaire[15]

^c^Cutoffs for model fit indices: CFI ≥ 0.95, RMSEA ≤ 0.08, SRMR ≤ 0.08

*Note*: All factor loadings are significant at *p* < .001

**Table 6:** Anticipated reactions to AD biomarker disclosure scale

Behavior change	
*Prompt:* I’m going to tell you some reasons why someone might want to learn their brain marker results. Please rate how important each of these are to you, as a reason to learn your brain marker results	
***Planning***^**a**^	
1.To know more about your risk of developing Alzheimer’s. **How important would this be to you as a reason to learn your brain marker results?**	< 1 > Not at all < 2 > A little < 3 > Somewhat < 4 > Very < 5 > Extremely
2.To inform lifestyle changes you might make, such as diet or exercise, that might help prevent Alzheimer’s. **How important would this be to you (as a reason to learn your brain marker results)?**	< 1 > Not at all < 2 > A little < 3 > Somewhat < 4 > Very < 5 > Extremely
3.To put your mind at ease if you found out your brain marker results did not indicate a higher risk for developing Alzheimer’s. **How important would this be to you?**	< 1 > Not at all < 2 > A little < 3 > Somewhat < 4 > Very < 5 > Extremely
4.To be able to participate in clinical trials attempting to lower brain marker results. **How important would this be to you?**	< 1 > Not at all < 2 > A little < 3 > Somewhat < 4 > Very < 5 > Extremely
5.To arrange your personal affairs, such as insurance, your will, or your finances. **How important would this be to you?**	< 1 > Not at all < 2 > A little < 3 > Somewhat < 4 > Very < 5 > Extremely
6.To confirm the feeling that you might already be developing symptoms of Alzheimer’s. **How important would this be to you?**	< 1 > Not at all < 2 > A little < 3 > Somewhat < 4 > Very < 5 > EXtremely
7.To prepare your family for your possible illness in the future. **How important would this be to you?**	< 1 > Not at all < 2 > A little < 3 > Somewhat < 4 > Very < 5 > Extremely
8.A desire to start doing things sooner than you had planned. **How important would this be to you?**	< 1 > Not at all < 2 > A little < 3 > Somewhat < 4 > Very < 5 > Extremely
*Prompt:* I want you to imagine that you participated in a study and were told that your brain marker results were high. While these results do not necessarily mean you will develop Alzheimer’s, it means you have a higher risk of developing Alzheimer’s	
***Risk-reduction***	
9.If you learned that your brain marker results were high and you were at higher risk of developing Alzheimer’s, how likely would you be to make changes in the following areas specifically to lower your risk of Alzheimer’s? **How likely would you be to make changes to your diet?**	< 1 > Not at all < 2 > A little < 3 > Somewhat < 4 > Very < 5 > Extremely
10.(If you learned that your brain marker results were high and you were at higher risk of developing Alzheimer’s) **How likely would you be to exercise or do more physical activity?**	< 1 > Not at all < 2 > A little < 3 > Somewhat < 4 > Very < 5 > Extremely
11.(If you learned that your brain marker results were high and you were at higher risk of developing Alzheimer’s) **How likely would you be to make changes to your medications?**	< 1 > Not at all < 2 > A little < 3 > Somewhat < 4 > Very < 5 > Extremely
12.(If you learned that your brain marker results were high and you were at higher risk of developing Alzheimer’s) **How likely would you be to make changes to or start taking vitamins or herbal supplements?**	< 1 > Not at all < 2 > A little < 3 > Somewhat < 4 > Very < 5 > Extremely
13.(If you learned that your brain marker results were high and you were at higher risk of developing Alzheimer’s) **How likely would you be to engage in activities to reduce stress?**	< 1 > Not at all < 2 > A little < 3 > Somewhat < 4 > Very < 5 > Extremely
14.(If you learned that your brain marker results were high and you were at higher risk of developing Alzheimer’s) **How likely would you be to engage in mental or cognitive activities such as crossword puzzles or Sudoku?**	< 1 > Not at all < 2 > A little < 3 > Somewhat < 4 > Very < 5 > Extremely
**Psychological impact**	
***Distress***	
15.If you learned that your brain marker results were high and you were at higher risk of developing Alzheimer’s **how upset would you feel about your results?**	< 1 > Not at all < 2 > A little < 3 > Somewhat < 4 > Very < 5 > Extremely
16.(If you learned that your brain marker results were high and you were at higher risk of developing Alzheimer’s) **How sad would you feel about your results?**	< 1 > Not at all < 2 > A little < 3 > Somewhat < 4 > Very < 5 > Extremely
17.(If you learned that your brain marker results were high and you were at higher risk of developing Alzheimer’s) **How anxious or nervous would you feel about your results?**	< 1 > Not at all < 2 > A little < 3 > Somewhat < 4 > Very < 5 > Extremely
18.(If you learned that your brain marker results were high and you were at higher risk of developing Alzheimer’s) **How worried would you be about your risk of developing Alzheimer’s?**	< 1 > Not at all < 2 > A little < 3 > Somewhat < 4 > Very < 5 > Extremely
19.(If you learned that your brain marker results were high and you were at higher risk of developing Alzheimer’s) **How uncertain would you be about what your results mean about your risk of developing Alzheimer’s?**	< 1 > Not at all < 2 > A little < 3 > Somewhat < 4 > Very < 5 > Extremely
20.(If you learned that your brain marker results were high and you were at higher risk of developing Alzheimer’s) **How uncertain would you be about what your results mean for your children or family’s risk of Alzheimer’s?**	< 1 > Not at all < 2 > A little < 3 > Somewhat < 4 > Very < 5 > Extremely
21.(If you learned that your brain marker results were high and you were at higher risk of developing Alzheimer’s) **How frustrated would you feel about there being no definite Alzheimer’s prevention guidelines for you?**	< 1 > Not at all < 2 > A little < 3 > Somewhat < 4 > Very < 5 > Extremely
22.(If you learned that your brain marker results were high and you were at higher risk of developing Alzheimer’s) **How hopeless would you feel about the future?**	< 1 > Not at all < 2 > A little < 3 > Somewhat < 4 > Very < 5 > Extremely
***Stigma***	
23.(If you learned that your brain marker results were high and you were at higher risk of developing Alzheimer’s) **How concerned would you feel about how your results might affect your insurance status?**	< 1 > Not at all < 2 > A little < 3 > Somewhat < 4 > Very < 5 > Extremely
24.(If you learned that your brain marker results were high and you were at higher risk of developing Alzheimer’s) **How concerned would you feel about the possibility that your doctor might learn the results and treat you differently?**	< 1 > Not at all < 2 > A little < 3 > Somewhat < 4 > Very < 5 > Extremely
25.(If you learned that your brain marker results were high and you were at higher risk of developing Alzheimer’s) **How concerned would you be that if others, such as family, friends, or co-workers, learned the results, they would act differently toward you?**	< 1 > Not at all < 2 > A little < 3 > Somewhat < 4 > Very < 5 > Extremely
***Subjective cognitive complaints***	
26.(If you learned that your brain marker results were high and you were at higher risk of developing Alzheimer’s) **How likely is it that you would have more trouble concentrating on things, such as reading the newspaper or watching television?**	< 1 > Not at all < 2 > A little < 3 > Somewhat < 4 > Very < 5 > Extremely
27.(If you learned that your brain marker results were high and you were at higher risk of developing Alzheimer’s) **How likely is it that you would have more trouble remembering things, such as recent conversations or events?**	< 1 > Not at all < 2 > A little < 3 > Somewhat < 4 > Very < 5 > Extremely
28.(If you learned that your brain marker results were high and you were at higher risk of developing Alzheimer’s) **How likely is it that you would be less confident about your ability to carry out daily activities?**	< 1 > Not at all < 2 > A little < 3 > Somewhat < 4 > Very < 5 > Extremely

*Footnote*: ^a^Items adapted from “Views and Perceptions of Amyloid Imaging” Questionnaire [15]

### Measurement invariance testing across racial groups

As shown in Table 5, the two-factor Behavioral Change model and three-factor Psychological Impact model confirmed in the total sample fit well when tested separately for Black and White participants. Configural, metric, and scalar invariance were next tested using the two-group CFAs and supported by good model fit indices and minimal change in CFI (see Table 7).

**Table 7:** Measurement invariance testing with two-group confirmatory factor analysis

	χ^2^ test			Model fit			Model comparison	
	χ^2^	df	*p* value	CFI	RMSEA (90% CI)	SRMR		∆CFI
Behavior Change Scale								
1.Configural	209.276	144	< 0.001	0.967	0.059 (0.040, 0.076)	0.049		
2.Metric	231.597	158	< 0.001	0.963	0.060 (0.043, 0.076)	0.062	1 vs 2	0.004
3.Scalar	262.033	170	< 0.001	0.954	0.064 (0.048, 0.079)	0.067	2 vs 3	0.009
Psychological Impact Scale								
1.Configural	204.775	138	< 0.001	0.970	0.056 (0.039, 0.072)	0.052		
2. Metric	212.912	149	< 0.001	0.971	0.053 (0.036, 0.068)	0.057	1 vs 2	-0.001
3. Scalar	248.344	160	< 0.001	0.961	0.060 (0.045, 0.074)	0.063	2 vs 3	0.010

## Discussion

As AD clinical trials continue to recruit at-risk middle-aged and older adults, it will become increasingly common for adults without symptoms to learn they have AD pathologic brain changes or preclinical AD. We need tools to identify factors associated with willingness to enroll in biomarker research to support clinical trial enrollment. Ideally, tools should assess a range of outcomes associated with learning AD biomarker results. We developed and administered a telephone survey to 334 middle-aged and older research participants to assess their willingness to learn AD biomarker results and anticipated reactions to learning results. Exploratory and confirmatory factor analysis revealed an underlying factor structure comprising anticipated outcomes of behavior change and psychological impact following disclosure of positive AD biomarker results.

The items included in the Behavior Change scale comprised two factors assessing (1) planning and (2) risk-reduction. The *planning* factor items were adapted from an existing scale originally administered in the REVEAL *APOE* disclosure study. In the REVEAL study of 206 adult children of people with AD, the best predictor of pursuing testing was a strong endorsement of the need to prepare family members should the participant develop AD dementia [14]. A separate study using this scale in a sample of at-risk adults enrolled in the Alzheimer’s Prevention Registry found the most common reasons for wanting to learn results were to participate in AD research, arrange personal affairs, and move plans closer in the future [6]. In our sample, the two most commonly endorsed reasons for learning AD biomarker results included to (1) inform lifestyle changes such as diet or exercise that might help prevent AD and (2) put mind at ease if found out results did not indicate a higher risk for developing AD. The *risk-reduction* factor included six items assessing respondents’ likelihood of changing lifestyle behaviors to reduce dementia risk after learning a positive AD biomarker result. A majority of participants endorsed a high likelihood of making various lifestyle changes if they learned their biomarker results were positive. These results are important because they suggest that despite no currently available preclinical AD treatment, people report anticipated personal utility in learning biomarker results. Specifically, these results demonstrate that people want to proactively reduce their risk for dementia. Disclosure of an elevated/positive AD biomarker result in particular may be useful in informing and potentially motivating people to address modifiable risk factors for dementia. Going forward, high scores on the planning or risk-reduction factors may be used to anticipate potential behavior changes to reduce dementia risk after learning high-risk results.

The items included in the Psychological Impact scale comprised three factors assessing (1) distress, (2) stigma, and (3) cognitive symptoms. Studies conducting AD biomarker disclosure have found a transient increase in test-related distress following results disclosure [27, 28]. However, these initial elevations in distress appear to remit over time, suggesting disclosure does not likely lead to long-term psychological symptoms. The *cognitive symptoms* factor included items assessing potential increased difficulties with concentration, memory, or confidence in carrying out daily activities. Increased subjective memory complaints and poorer objective cognitive performance have been shown in cognitively unimpaired adults after learning their genetic risk for AD (*APOE* ε4 carrier result)[29]. More recent studies suggest increased subjective memory symptoms upon learning of a positive amyloid PET scan result [4]. The *stigma* factor included items assessing concern about how learning AD biomarker results might affect insurance status or treatment from doctors or family, friends, and co-workers. Emerging evidence suggests that stigma associated with dementia may also be experienced by those not yet diagnosed including social isolation, discrimination, and internalized distress [3]. High scores on the psychological impact scale may suggest a higher risk of psychological symptoms following disclosure and need for additional supports. Overall, participant education about the transient nature of psychological distress and additional provision of support to mitigate the psychological impact of learning high-risk results may be beneficial in development of disclosure protocols.

We focused our recruitment efforts to ensure representation of adults who identified as Black or African American in our study as AD research is significantly limited by its use of predominantly White samples [30]. Understanding racial differences in willingness to enroll in AD biomarker studies and potential reactions to learning high-risk results can support development of culturally-informed AD biomarker disclosure practices. In tests of measurement invariance, the factor structure above was confirmed across race at the scalar invariance level, which allows for meaningful comparisons of latent factor means, variances, and correlations across racial groups. This is important to ensure similar interpretation of the meaning of the results of this scale across Black and White participants in our study. In examining willingness to enroll in biomarker studies, we previously reported that Black participants were less willing than White participants, but that willingness increased if results were shared back to participants [31]. This result suggests that learning results is particularly important to the Black participants in our cohorts, an example of the growing literature on the importance of building transparency and trust with marginalized populations [32]. Although there are additional studies on factors that influence clinical research enrollment in Black participants [33–36], there is a lack of published data on hypothetical or actual reactions to learning AD biomarker results in Black participants. Our next steps include to evaluate relationships among contextual factors assessed in this survey (e.g., research attitudes, perceived discrimination, concern about and experience with AD) with anticipated reactions to learning biomarker results.

Important limitations to the current study should be considered. The survey was administered to a convenience sample of adults already enrolled in AD research, and at higher than average risk for ADRD. Although this population is relevant as these individuals are likely to be targeted for early-stage clinical trials, this means our results likely do not readily generalize to the general population. In addition to being more familiar with and at risk for AD, these participants were highly educated. Interest in and anticipated reactions to learning biomarker results may be different in less educated populations in which more misunderstanding about biomarker testing and results may occur. Future studies could use a population-based approach or otherwise involve the general population to better understand reactions of those less familiar with AD or research studies and those with fewer years of education. Assessment and inclusion of information about socioeconomic status, such as income and occupation, in addition to education, will also be important for future studies to fully characterize contextual factors that may influence willingness to learn results and anticipated reactions. Additionally, results from this survey are based on anticipated reactions to hypothetical scenarios, not actual responses to learning biomarker results; as noted in previous studies on diminished interest to learn biomarker results with education about their meaning and utility, it is possible that reactions to learning results may vary with more education about meaning of results. Further, changes in availability of treatment options will likely influence people’s anticipated reactions.

## Conclusions

Understanding participant willingness to enroll in AD biomarker studies and anticipated reactions to learning biomarker results is important for improving participation and informing study development, particularly when considering whether to return biomarker results. The Alzheimer’s Biomarker Survey included items assessing willingness to learn biomarker results and anticipated reactions to learning hypothetical results including planning, dementia risk reduction, distress, cognitive symptoms, or stigma. Examination of these responses along with additional beliefs about AD, research, health, and perceived discrimination in diverse populations can provide a foundation for education, disclosure, and post-disclosure guidelines to improve individual experiences following AD biomarker disclosure.

## Data Availability

The datasets used and/or analyzed during the current study are available from the corresponding author on reasonable request.

## References

[1] Cummings J, Aisen P, Apostolova LG, Atri A, Salloway S, Weiner M (2021). Aducanumab: appropriate use recommendations. J Prev Alzheimers Dis.

[2] Zetterberg H, Burnham SC (2019). Blood-based molecular biomarkers for Alzheimer’s disease. Mol Brain.

[3] Stites SD, Milne R, Karlawish J (2018). Advances in Alzheimer’s imaging are changing the experience of Alzheimer’s disease. Alzheimers Dement Diagn Assess Dis Monit.

[4] Largent EA, Harkins K, van Dyck CH, Hachey S, Sankar P, Karlawish J (2020). Cognitively unimpaired adults’ reactions to disclosure of amyloid PET scan results. PLoS ONE.

[5] Gooblar J, Roe CM, Selsor NJ, Gabel MJ, Morris JC (2015). Attitudes of research participants and the general public regarding disclosure of Alzheimer disease research results. Jama Neurol.

[6] Ott BR, Pelosi MA, Tremont G, Snyder PJ (2016). A survey of knowledge and views concerning genetic and amyloid positron emission tomography status disclosure. Alzheimers Dement Transl Res Clin Interv.

[7] Milne R, Bunnik E, Diaz A, Richard E, Badger S, Gove D (2018). Perspectives on communicating biomarker-based assessments of Alzheimer’s disease to cognitively healthy individuals. J Alzheimers Dis.

[8] Vanderschaeghe G, Vandenberghe R, Dierickx K (2019). Stakeholders’ views on early diagnosis for Alzheimer’s disease, clinical trial participation and amyloid PET disclosure: a focus group study. J Bioethical Inq.

[9] Roberts JS, Connell CM (2000). Illness representations among first-degree relatives of people with Alzheimer disease. Alzheimer Dis Assoc Disord.

[10] Wallston KA, Wallston BS, DeVellis R (1978). Development of the Multidimensional Health Locus of Control (MHLC) scales. Health Educ Monogr.

[11] Seifan A, Ganzer CA, Vermeylen F, Parry S, Zhu J, Lyons A (2017). Development and validation of the Alzheimer’s prevention beliefs measure in a multi-ethnic cohort-a behavioral theory approach. J Public Health Oxf Engl.

[12] Rubright JD, Cary MS, Karlawish JH, Kim SYH (2011). Measuring how people view biomedical research: reliability and validity analysis of the Research Attitudes Questionnaire. J Empir Res Hum Res Ethics.

[13] Williams DR, Yan Y, Jackson JS, Anderson NB (1997). Racial differences in physical and mental health: socio-economic status stress and discrimination. J Health Psychol.

[14] Roberts JS, LaRusse SA, Katzen H, Whitehouse PJ, Barber M, Post SG (2003). Reasons for seeking genetic susceptibility testing among first-degree relatives of people with Alzheimer disease. Alzheimer Dis Assoc Disord.

[15] Ryan MM, Gillen DL, Grill JD (2021). Reasons for undergoing amyloid imaging among cognitively unimpaired older adults. Ann Clin Transl Neurol.

[16] Mckhann GM, Knopman DS, Chertkow H, Hyman BT, Jack CR, Kawas CH (2011). The diagnosis of dementia due to Alzheimer’s disease: Recommendations from the National Institute on Aging-Alzheimer’s Association workgroups on diagnostic guidelines for Alzheimer’s disease. Alzheimers Dement J Alzheimers Assoc.

[17] Albert MS, DeKosky ST, Dickson D, Dubois B, Feldman HH, Fox NC (2011). The diagnosis of mild cognitive impairment due to Alzheimer’s disease: recommendations from the National Institute on Aging-Alzheimer’s Association workgroups on diagnostic guidelines for Alzheimer’s disease. Alzheimers Dement J Alzheimers Assoc.

[18] Muthén B (1984). A general structural equation model with dichotomous, ordered categorical, and continuous latent variable indicators. Psychometrika.

[19] Bentler PM (1990). Comparative fit indexes in structural models. Psychol Bull.

[20] Steiger JH (1980). Statistically based tests for the number of common factors.

[21] Browne MW, Cudeck R (1992). Alternative Ways of Assessing Model Fit. Sociol Methods Res..

[22] Hu L, Bentler PM (1999). Cutoff criteria for fit indexes in covariance structure analysis: Conventional criteria versus new alternatives. Struct Equ Model Multidiscip J.

[23] Hu L, Bentler PM (1998). Fit indices in covariance structure modeling: Sensitivity to underparameterized model misspecification. Psychol Methods US: Am Psychol Assoc.

[24] Revelle W. psych: Procedures for Psychological, Psychometric, and Personality Research. Northwestern University, Evanston, Illinois; 2021. Available from: https://CRAN.R-project.org/package=psych.

[25] Rosseel Y (2012). lavaan: An R Package for Structural Equation Modeling. J Stat Softw.

[26] Cheung GW, Rensvold RB (2002). Evaluating Goodness-of-Fit Indexes for Testing Measurement Invariance. Struct Equ Model Multidiscip J Routledge.

[27] Burns JM, Johnson DK, Liebmann EP, Bothwell RJ, Morris JK, Vidoni ED (2017). Safety of disclosing amyloid status in cognitively normal older adults. Alzheimers Dement.

[28] Grill JD, Raman R, Ernstrom K, Sultzer DL, Burns JM, Donohue MC (2020). Short-term psychological outcomes of disclosing amyloid imaging results to research participants who do not have cognitive impairment. JAMA Neurol.

[29] Lineweaver TT, Bondi MW, Galasko D, Salmon DP (2014). Effect of knowledge of APOE genotype on subjective and objective memory performance in healthy older adults. Am J Psychiatry.

[30] Gleason CE, Zuelsdorff M, Gooding DC, Kind AJH, Johnson AL, James TT, et al. Alzheimer’s disease biomarkers in Black and non-Hispanic White cohorts: a contextualized review of the evidence. Alzheimers Dement J Alzheimers Assoc. 2021. 10.1002/alz.12511. Epub ahead of print. 10.1002/alz.12511 PMC9543531 34870885

[31] Erickson CM, Chin NA, Ketchum FB, Jonaitis EM, Zuelsdorff ML, Gleason CE (2022). Predictors of willingness to enroll in hypothetical Alzheimer disease biomarker studies that disclose personal results. Alzheimer Dis Assoc Disord..

[32] Dave G, Frerichs L, Jones J, Kim M, Schaal J, Vassar S (2018). Conceptualizing trust in community-academic research partnerships using concept mapping approach: a multi-CTSA study. Eval Program Plann.

[33] Zhou Y, Elashoff D, Kremen S, Teng E, Karlawish J, Grill JD (2016). African Americans are less likely to enroll in preclinical Alzheimer’s disease clinical trials. Alzheimers Dement Transl Res Clin Interv.

[34] Luebbert R, Perez A (2016). Barriers to Clinical Research Participation Among African Americans. J Transcult Nurs Off J Transcult Nurs Soc.

[35] Williams MM, Scharff DP, Mathews KJ, Hoffsuemmer JS, Jackson P, Morris JC (2010). Barriers and facilitators of African American participation in Alzheimer disease biomarker research. Alzheimer Dis Assoc Disord.

[36] Shavers VL, Lynch CF, Burmeister LF (2002). Racial differences in factors that influence the willingness to participate in medical research studies. Ann Epidemiol.

